# Logistic regression prediction model identify type 2 diabetes mellitus as a prognostic factor for human papillomavirus-16 associated head and neck squamous cell carcinoma

**DOI:** 10.1371/journal.pone.0217000

**Published:** 2019-05-16

**Authors:** Aastha Sobti, Fatemeh Saheb Sharif-Askari, Saif Khan, Narjes Saheb Sharif-Askari, Mahmood Yaseen Hachim, Luke Williams, Yuanping Zhou, Colin Hopper, Rifat Hamoudi

**Affiliations:** 1 Academic Unit of Oral and Maxillofacial Surgery, UCL Eastman Dental Institute, London, United Kingdom; 2 Sharjah Institute of Medical Research, College of Medicine, University of Sharjah, Sharjah, United Arab Emirates; 3 Research Department of Targeted Intervention, Division of Surgery and Interventional Science, University College London, London, United Kingdom; 4 Department of Infectious Diseases and Hepatology Unit, Nanfang Hospital, Southern Medical University, Guangzhou, China; King's College London, UNITED KINGDOM

## Abstract

**Background:**

HPV-16–positive HNSCC and HPV-16–negative HNSCC have different clinical factors, representing distinct forms of cancers. The study aimed to identify patient-specific factors for HPV-16-positive HNSCC based on baseline clinical data.

**Method:**

Factors associated with HPV-16-positive HNSCC were identified using the data from 210 patients diagnosed with HNSCC at University College of London Hospital between January 1, 2003, and April 30, 2015, inclusive. A series of models were developed using logistic regression methods, and the overall model fit was compared using Akaike Information Criterion. Survival analysis was carried with Cox proportional hazards model for survival-time outcomes. The survival time for individual patients was defined as the time from diagnosis of HNSCC to the date of death from any cause. For patients who did not die, they were censored at the end of study on April 30, 2015.

**Results:**

Of the 210 patients, 151 (72%) were found to have HPV-16-positive HNSCC. The logistic regression model showed that the prevalence of developing HPV-16-positive HNSCC was 3.79 times higher in patients with Type 2 Diabetes Mellitus (T2DM) (odd ratio [OR], 3.79; 95% CI, 1.70–8.44) than in those without T2DM, and 8.84 times higher in patients with history of primary HNSCC (OR, 8.84; 95% CI, 2.30–33.88) than in those without a history of primary HNSCC. HPV-16–positive HNSCC was also observed more in tonsils (OR, 4.02; 95% CL, 1.56–10.36) and less in non-alcohol drinker’s oral cavity (OR, 0.14; 95% CI, 0.03–0.56). Furthermore, individual patients were followed-up for 1 to 13 years (median of 1 year). Patients with HPV-positive HNSCC had a median survival of 5 years (95% CI, 2.6–7.3 years). Among HPV-16–positive HNSCC cohort, T2DM was a risk for poorer prognosis (hazard ratio, 2.57; 95% Cl, 1.09–6.07), and had lower median survival of 3 years (95% CI, 1.8–4.1 years), as compared to 6 years (95% CI, 2.8–9.1 years) in non-T2DM.

**Conclusions:**

Patient-specific factors for HPV-positive HNSCC are T2DM, history of primary HNSCC and tonsillar site. T2DM is associated with poorer prognosis. These findings suggest that it might be beneficial if routine HPV-16 screening is carried out in T2DM patients which can provide better therapeutic and management strategies.

## Introduction

Over the past 3 decades, an increase in the incidence of human papillomavirus (HPV)-positive head and neck cancer (HNSCC), particularly oropharyngeal cancer, has been observed in the United Kingdom, most notably among men under the age of 60 [1]. Previous reports suggest that HPV-positive HNSCC and HPV-negative HNSCC are heterogeneous, representing distinct subtypes of HNSCC, each with different risk and prognostic factors [2–6].

Few studies developed risk predictive models of tumor HPV-16–status using tumor biomarkers [2] or histopathological features [4]. Features of a patient with HPV-16–positive HNSCC is younger onset, male predominance, limited or no prior smoking or alcohol use, white race and the presence of pathological factors such as oropharynx primary tumor, p16 protein overexpression, poor tumor differentiation, basaloid features, and non-keratinizing cell types on histopathologic analysis. However, currently there are no accepted predictive models to identify patient specific factors of HPV-16–positive HNSCC based on baseline demographic and clinical data. This may lead physicians to make ad hoc decisions about which patients to receive routine HPV-16 screening, and risk delay in testing those who are at risk of HPV-16–positive HNSCC development. Thus, establishing a link between patient clinical and medical history data such as co-morbidities and tumor HPV status can provide valuable insights as to which patients should undergo routine screening. Therefore, we developed a methodology to identify patient-specific factors for HPV-16–positive HNSCC based on baseline clinical and medical history data. We also followed-up individual patients for 1 to 13 years (median of 1 year), to identify which patient factors are associated with poorer prognosis.

## Materials and methods

### Ethical considerations

The study protocol was approved by the Institutional Review Board of the UCLH. The ethic number for the study is 04/Q0505/59 by National Research and Ethics Committee (NRES) London-Harrow through University College of London Hospitals NHS Foundation Trust. Patients’ records were fully anonymized prior to access.

### Study population

The study cohort was derived from the head and neck cancer department medical records at University College of London Hospital (UCLH). Patients diagnosed with HNSCC between January 1, 2003, and April 30, 2015, at UCLH were included, inclusive. Patients were eligible for inclusion if they were older than 18 years and were newly diagnosed with a histological confirmed squamous cell carcinoma of the oral cavity, oropharynx, tonsils, hypopharynx, larynx, nasopharynx, or multiple HNSCC tumor sites. Multiple HNSCC tumor sites was defined as the presence of two or more tumors synchronously in head and neck region. Anatomic site of the tumor was determined by a physical examination that was performed by the treating head and neck surgeon. SPSS file consisting of all patients’ parameters used in model prediction for the study is provided in S1 File.

### HPV-16 in situ hybridization

The outcome of interest was tumor HPV-16 status. Patients were classified as having a diagnosis of either HPV-16-positive or HPV-16-negative HNSCC based upon HPV-16 DNA detection in formalin-fixed and paraffin-embedded (FFPE) tumors using the *in situ* hybridization approach for biotinylated probes [7]. Tissue consists of triplicate tumor cores from corresponding FFPE blocks from all patients were constructed. The positive control was p16 IHC and the negative control was inflamed tonsil tissue. Specific staining of tumor cell nuclei for HPV-16 defined a positive tumor.

### Univariate analysis of variables

Patient independent variables consisted of demographic variables, including age and sex; co-morbid conditions, including type 2 diabetes mellitus (T2DM), hypertension, vascular diseases, previous primary HNSCC, history of current or former smoking, or alcohol use; treatments history, including use of anti-hypertensives, hypoglycemic agents, or anti-infective for systemic use; and HNSCC tumor anatomic sites. A patient’s co-morbid conditions and treatment history were categorized as present or absent at the time of HNSCC diagnosis. The anatomic sites of head and neck included in the analysis were oropharynx, tonsils, oral cavity, larynx, hypopharynx, paranasal sinus, and nasopharynx. In this study, a large number of our patients (36%) were diagnosed with HPV-positive tonsillar carcinoma; therefore, tonsils which is a part of the oropharynx anatomic site were analyzed separately. Oropharynx included tumor in soft palate and uvula.

All selected variables were tested for multi co-linearity to avoid any strong correlation between the variables. The presence of co-linearity was examined by the evaluation of variance inflation factors and magnitude of standard errors. Variables with more than 30% missing values were not included in the analysis. All other missing data were imputed using the multiple imputation technique using SPSS (version 21; SPSS, Inc., Chicago, IL).

### Multivariate analysis and model development

**Fig 1:**
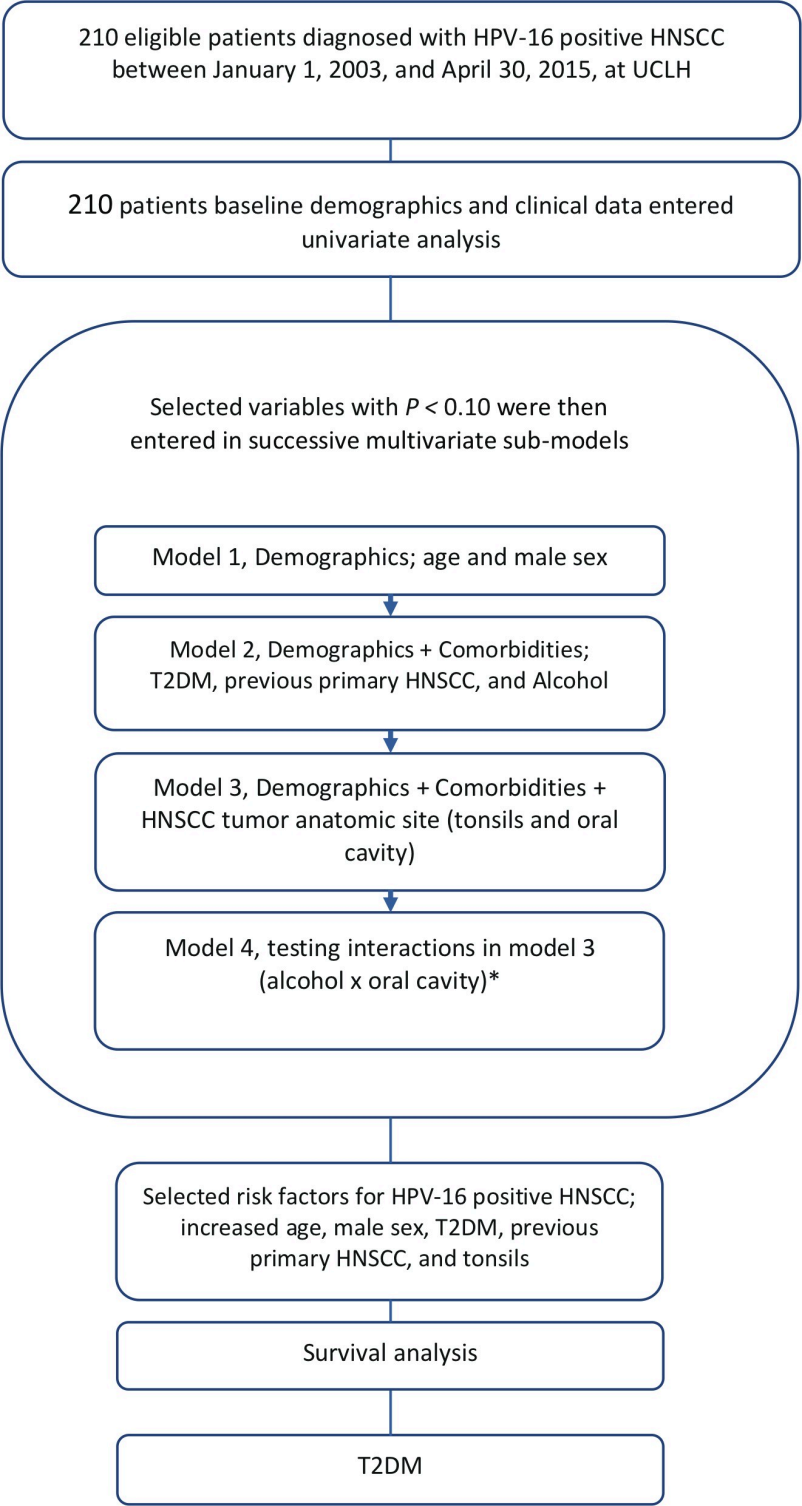
The flow chart of study methodology. Abbreviations: HNSCC, head and neck squamous cell carcinoma; HPV, human papillomavirus, T2DM; type 2 diabetes mellitus, UCLH, University College of London Hospital. *A significant interaction (*P* < .05) was identified between the oral cavity tumor site and alcohol use; therefore, to remove their simultaneous influence both were added to the final model.

The first aim of this study was to identify patient- specific factors associated with HPV-16–positive HNSCC based on baseline demographic and clinical data. Therefore, we developed a sequential series of logistic regression models using HPV-16–positive HNSCC as the dependent variable (Fig 1). We used a combination of clinical medical guidance and forward selection method to determine variable selection [8, 9]. Using univariate analysis, variables not associated with HPV-16–positive HNSCC (*P >*0.10) were excluded from further analyses. Variables were then entered in each model in accordance with the chronology in which patient demographic and clinical data were available at the time of HNSCC diagnosis. Model with more variables or with greater complexity was successively compared with the simple ones. Using this approach, 4 Models were generated from the data provided.

Model 1, was developed by adding patient demographics (age and male sex). Model 2, was developed by adding patient co-morbidities (T2DM, previous HNSCC, and alcohol use) and compared with model 1. Model 3, was developed by adding patient HNSCC tumor anatomic sites (tonsils and oral cavity) and compared with model 2. We then tested for interaction effects between predictors or variables retained in model 3. A significant interaction (*P* < 0.05) was identified between the oral cavity tumor site and alcohol use; therefore, both variables were added to the final model (Model 4).

### Model performance evaluation

Improvement in model performance through the addition of new variables in multivariate logistic regression models was tested using measures of discrimination and goodness of fit. The discriminatory power of each model was assessed using concordance statistics (C statistics). Discrimination refers to the ability of a model to clearly distinguish between 2 groups of outcomes (discriminate between the HPV-16–positive HNSCC and HPV-16–negative HNSCC patient groups) and can range from 0.5 (no discrimination) to 1.0 (perfect discrimination) [10, 11]. The overall model fit for sequential models was compared using Akaike Information Criterion (AIC), which considers both the statistical goodness of fit and the number of variables required to achieve this particular degree of fit by imposing a penalty for increasing the number of variables. The optimal fitted model was selected by the minimum value of AIC [11]. We used AIC over the BIC (Bayesian Information Criterion) because selection with BIC may lead to “under-fitting,” since BIC penalizes larger models by excluding many predictors [11]. The accuracy of the final model was evaluated using positive predictive value (percentage of truly positive among those predicted HPV-positive HNSCC), and negative predictive value (percentage of truly negative among those predicted HPV-positive HNSCC). Sensitivity (percentage predicted positive among all truly positive) and specificity (percentage predicted negative among all truly negative) were also calculated.

### Survival analysis

The second aim of this study was to compare the median survival time between patients with HPV-positive and HPV-negative tumors during the follow-up period of 1 to 13 years (median of 1 year). We also compared the median survival time between T2DM and non-T2DM subgroups within the HPV-positive HNSCC cohort. The survival time for individual patients was defined as the time from diagnosis of HNSCC to the date of death from any cause. For patients who did not die, they were censored at the end of study on April 30, 2015. Survival probability was computed with a Cox proportional hazards model and the resultant cumulative survival probability curve was plotted. Cox proportional hazards model was adjusted for age, male sex, alcohol use, smoking, and American Society of Anesthesiologist (ASA) co-morbidity score [12].

All analyses were performed using SPSS 21.0. Categorical variables are presented herein as percentages, and the continuous variable (age) are presented as means (SDs). The age variable was assessed for normality using the Kolmogorov-Smirnov test. Comparisons of categorical variables were performed using the chi-square (χ^2^) test or Fisher exact test. A finding of *P* < 0.05 was considered indicative of a statistically significant difference for all tests.

## Results

Following a review of the medical records, we determined that 210 patients were diagnosed with HNSCC at UCLH, 151 of which were HPV-16–positive HNSCC (72%). Consistent with recent epidemiological studies [1, 6, 13], almost two-third of the patients in this study had HPV-related tumors in oropharyngeal sites (100 cases out of 151, 66%). Of those 100 patients with HPV-16–related oropharyngeal cancer, 54 patients had carcinoma of the tonsillar regions (Table 1).

**Table 1:** Baseline characteristics of study cohort.

	No. (%) of Patients		
Characteristics	HPV-16–positive HNSCC (n = 151)	HPV-16–negative HNSCC (n = 59)	*P* value
**Demographic**			
Age, mean (SD), y	61 (12)	61 (11)	0.742
Male sex	112 (74)	37 (63)	0.100
**Co-morbidities**			
Hypertension	17 (11)	5 (8)	0.554
Cardiovascular diseases	29 (19)	11 (19)	0.926
T2DM	68 (45)	16 (27)	0.017
Previous malignancies	62 (41)	30 (51)	0.218
Previous primary HNSCC	27 (18)	4 (7)	0.042
History of current or former smoker	109 (72)	40 (68)	0.529
Alcohol use	117 (77)	29 (49)	<0.001
Smokeless tobacco	6 (4)	4 (7)	0.391
**Treatment history**			
Antihypertensive	16 (11)	2 (3)	0.107
Hypoglycemic agents	9 (6)	3 (5)	0.806
Antidepressants	6 (4)	1 (2)	0.408
Anti-infective for systemic use	6 (4)	6 (10)	0.101
**HNSCC anatomic sites**			
Oral cavity	29 (19)	22 (37)	0.006
Oropharynx	37 (24)	9 (15)	0.145
Tonsils	54 (36)	7 (12)	0.001
Multiple HNSCC sites	24 (16)	6 (10)	0.287
Other HNSCC sites*	7 (5)	15 (25)	<0.001

Abbreviations: HNSCC, head and neck squamous cell carcinoma; HPV, human papillomavirus, T2DM; type 2 diabetes mellitus.

*Other HNSCC tumor sites included tumors in larynx (n = 10), or hypopharynx (n = 5), or nasopharynx (n = 4), or paranasal sinus (n = 3).

### Univariate analysis of HNSCC variables

The patient demographic and clinical characteristics captured in the database are listed in Table 1. The mean age was similar between the HPV-16–positive and HPV-16–negative patient groups (61 years), with more males in the HPV-16–positive compared to HPV-16–negative patient groups (74% vs 63%, *P* = 0.100). Compared to HPV-16–negative patient groups, those with HPV-16–positive HNSCC had significantly higher number of co-morbidities such as T2DM (45% vs 27%, *P* = 0.017), history of primary HNSCC (18% vs 7%, *P* = 0.042), or alcohol use history (77% vs 49%, *P*<0.001); and had significantly higher and lower rates of HNSCC in tonsils (36% vs 12%, *P*<0.001) and oral cavity (19% vs 37%, *P*<0.006), respectively.

### Multivariate analysis of HNSCC data using statistical model development

The odd ratios for the variables and statistics for discrimination and goodness of fit for successive models are shown in Table 2. Model 1, including age and sex only, performed poorly (C statistic, 0.563; 95% confidence interval [CI], 0.476–0.650, and AIC 250.57; P< 0.001). However, the C statistic and AIC improved with the inclusion of patient co-morbidities such as T2DM, history of primary HNSCC, and alcohol use in model 2 (0.760; 95% CI, 0.689–0.832, and 222.17; P< 0.001), and HNSCC anatomic sites such as tonsils and oral cavity in model 3 (0.800; 95% CI, 0.732–0.868, and 211.97; P< 0.001). After the addition of interaction terms between alcohol use and oral cavity in model 4, test results were significantly associated with HPV-16–positive HNSCC for presence of T2DM, history of primary HNSCC, and HNSCC sites in tonsils and non-alcohol drinker’s oral cavity (0.825; 95% CI, 0.761–0.889, and 203.51; P< 0.001). The final model showed a good predictive ability for tumor HPV-16–status (n = 210, sensitivity (%) of 81, specificity (%) of 76, positive predictive value (%) of 94, and negative predictive value (%) of 42).

**Table 2:** Odd ratios and goodness of fit for sequential models to predict a HPV-16–positive HNSCC.

	Models			
Variables	1	2	3	4
	Demographic	Demographic + Co-morbid conditions	Demographic + Co-morbid conditions + HNCSS anatomic sites	Demographic + Co-morbid conditions HNCSS anatomic locations + Effect modifications**
Age, 1-y increase	0.99 (0.96–1.02)	1.00 (0.97–1.03)	1.00 (0.97–1.03)	1.01 (0.97–1.04)
Male sex	1.72 (0.90–3.29)	1.54 (0.75–3.14)	1.50 (0.71–3.14)	1.46 (0.68–3.15)
T2DM		3.36 (1.61–6.99)	3.67 (1.70–7.91)	3.79(1.70–8.44)
Previous primary HNSCC		6.67 (2.00–22.23)	7.86 (2.30–26.82)	8.84 (2.30–33.88)
Alcohol		4.32 (2.14–8.73)	4.15 (1.97–8.73)	1.94 (0.79–4.74)
Tonsils			4.18(1.58–11.05)	4.02(1.56–10.36)
Oral cavity			0.68 (0.31–1.52)	0.14 (0.03–0.56)
Alcohol x Oral cavity				15.85 (2.63–95.20)
C statistic*	0.563 (0.476–0.650)	0.760 (0.689–0.832)	0.800 (0.732–0.868)	0.825 (0.761–0.889)
Akaike* Information Criterion	250.57	222.17	211.97	203.51
*P* value	< .001	< .001	< .001	< .001

Abbreviations: HNSCC, head and neck squamous cell carcinoma, T2DM; type 2 diabetes mellitus.

Data are presented as odd ratios (95% confidence interval) unless otherwise specified.

* Higher values for C statistic and lower values for Akaike Information Criterion indicate better models.

** A significant interaction (*P* < .05) was identified between the oral cavity tumor site and alcohol use; therefore, to remove their simultaneous influence both were added to the final model (model 4). The resultant OR for the oral cavity tumor site is specific for non-alcohol drinkers.

### Survival analysis

**Fig 2:**
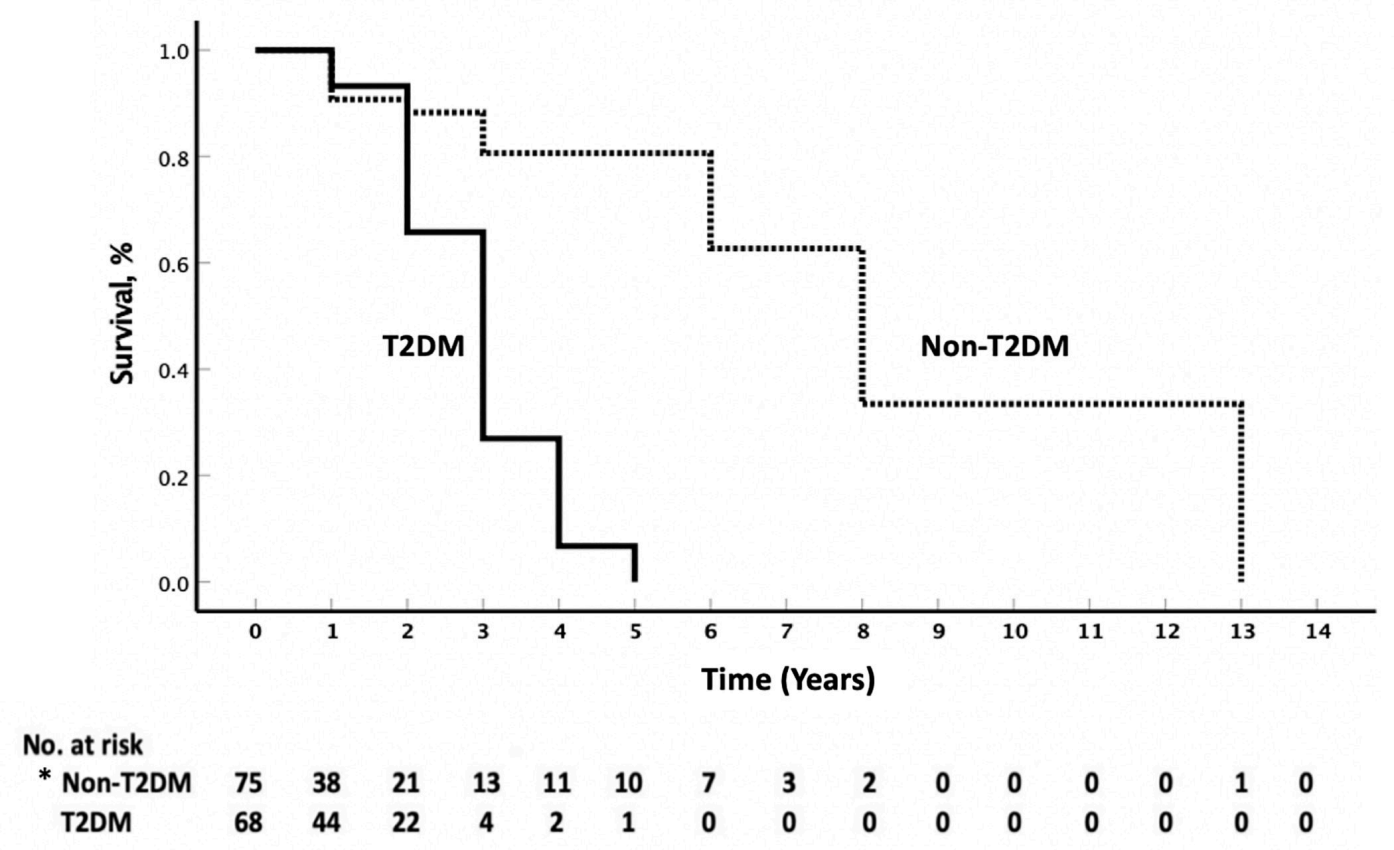
Cumulative survival probability of patients with T2DM or non-T2DM human papillomavirus-16 positive HNSCC. *The T2DM status for 8 patients with HPV-positive HNSCC was unknown. Abbreviations: HNSCC, head and neck squamous cell carcinoma; HPV, human papillomavirus, T2DM; type 2 diabetes mellitus.

Individual patients were followed-up for 1 to 13 years (median of 1 year), during which time 47 deaths occurred. Patients with HPV-positive HNSCC had a median survival of 5 years (95% CI, 2.6–7.3 years). HPV-positive HNSCC cohort with T2DM and a history of previous HNSCC, or with diagnosis of tonsillar or oral cavity were further stratified. A multivariate Cox proportional hazards regression analysis, adjusted for sex, age, alcohol use, smoking, and ASA co-morbidity score, revealed that among above-mentioned HPV-positive HNSCC specific factors, T2DM were a risk for poorer prognosis (hazard ratio, 2.57; 95% Cl, 1.09–6.07), and had lower median survival of 3 years (95% CI, 1.8–4.1 years), as compared to 6 years (95% CI, 2.8–9.1 years) in non-T2DM. (Table 3 and Fig 2).

**Table 3:** Summary of survival outcome of patients with HPV-16 positive HNSCC.

	Median survival rate in years (95% CI)	HR (95% CI*)	*P*-value*
**A. Survival**			
HPV-16 negative HNSCC	5	1	
HPV-16 positive HNSCC	5 (2.6–7.3)	1.08 (0.46–2.51)	0.851
**B. Survival in Subgroups with HPV-16 positive HNSCC**			
Non-T2DM	6 (2.8–9.1)	1	
T2DM	3 (1.8–4.1)	2.57 (1.09–6.07)	0.031
Non-previous primary HNSCC	4 (1.3–6.6)	1	
Previous primary HNSCC	6 (2.3–9.6)	0.68 (0.19–2.33)	0.542
Non-tonsil	4 (2.7–5.2)	1	
Tonsil	13	0.39 (0.13–1.20)	0.104
Non-oral cavity	4 (2.1–5.8)	1	
Oral cavity	7	0.80 (0.26–2.44)	0.70

*P-value are adjusted for age, male sex, alcohol, smoking, and ASA comorbidity score

## Discussion

In this study, we developed a series of risk prediction models in a cohort of HNSCC to identify patient specific factors associated with tumor HPV-16 status. A comprehensive clinical predictive model would serve to help clinicians decide which patient-factor is associated with higher prevalence of HPV-16–positive HNSCC. As there is a different factors associated with tumor HPV-16–status, we also followed-up individual patients for 1 to 13 years (median of 1 year), and found that there is also heterogeneity in the prognostic factors associated with tumor HPV-16-status.

Several prior studies have developed prediction models from several likely patient-related factors in patients with HNSCC [2, 4].Byrd et al [4] showed that a multivariable model including socio-demographic and histopathological factors was moderately successful at predicting HPV-16-status among HNSCC. The only variables significantly related to HPV-16-status were male sex (*P* = .01) and oropharyngeal subsite (*P* = 0.02). Similarly, D’Souza et al [2] found that models that used a combination of demographic characteristics such as age, tobacco use, male sex, and race had only moderate predictive value for tumor HPV-16–status among all patients with HNSCC (n = 225, sensitivity (%) of 76 and specificity (%) of 60) or when limited to oropharynx cancer patients (n = 119, sensitivity (%) of 84 and specificity (%) of 31). Addition of behavioral factors such as lifetime number of sexual partners, family income, education; or biomarkers of HPV-16 exposure such as L1, E6/7 antibodies or DNA in oral exfoliated cells to the multivariate model did not improve prediction. Nevertheless, in our study, variables were added in each model in accordance with the chronology in which patient demographic and clinical data were available at the time of HNSCC diagnosis. The final model showed a good predictive ability for tumor HPV-16 status (n = 210, the sensitivity of 81% and specificity of 76%). The advantage of our method is in its simplicity and potentially using more patient-centered approach enabling clinicians to identify high-risk groups of patients for HPV-16 status who may benefit from preventive measures such as routine screening for oral HPV-16 infection, thereby minimizing their risk of HPV-16related HNSCC [14].

In this study, we found that patients who had a history of primary HNSCC were significantly at higher risk of recurrent second primary HPV-16–positive HNSCC. Our finding correlates with previous studies that have reported a trend between previous HNSCC and second primary HNSCC in an era of oncogenic HPV [15, 16]. Diaz et al. [16] performed a study in Surveillance, Epidemiology, and End Results (SEER) database (1973–2008) for 104,639 patients with an index HNSCC, and found that 4616 (4.4%) had second primary HPV-positive oral cavity/pharyngeal cancers. The author concluded that there is a shift in HNSCC etiology to a tumor primarily caused by oncogenic HPV over tobacco associated HNSCC. Now, the question that arises is whether persistent HPV infection in the head and neck region is a risk factor for pharyngeal cancer which hasn’t been rigorously explained [17]. However, certain groups of patients with index HNSCC may likely benefit from routine HPV-16 screening measures [14, 18] making our model potentially useful for further research as we identified high-risk patients for HPV-16–positive HNSCC development.

A noteworthy finding in our study was that patients with T2DM were significantly associated with higher prevalence of HPV-16–positive HNSCC and were associated with poorer prognosis in HPV-16-positive HNSCC subgroups. Numerous epidemiological studies have reported a higher risk of oral precancerous lesions [19] and oropharyngeal cancer in patients with DM [20, 21], and some also revealed that DM are correlated with poorer prognosis[20, 22]; however, these studies were unable to control for tumor HPV status. One possible explanation that relates T2DM to HPV-associated HNSCC, is that DM (particularly if glycemic control is poor, HbA1c >9%) is associated with an increased prevalence of periodontitis [23], and, chronic periodontitis is associated with HPV-positive oropharyngeal cancer [24]. Periodontitis is a chronic oral infection caused by inflammatory reactions to gram-negative anaerobic bacteria in the dental plaque [25]. It results in continuous release of inflammatory cytokines, including interleukins (IL)-1, IL-6, and tumor necrosis factor-α, that modulate proliferation of HPV and expression of its oncogene E6/E7 in epithelial cells [26]. Subsequently, the expression of the E6/E7 oncogenes can lead to early compromise of the innate immune system (loss of antigen-presenting cells) promoting HPV persistence and increased risk of cancer [27]. The periodontal pocket may also act as a reservoir for latent HPV [28].Thus, another reasonable explanation that links T2DM to the development of HNSCC may be their long-term exposure to HPV. Most HPV infections are cleared rapidly by an intact immune system and do not lead to cancer. But the lower immune response of DM [29] facilitates persistence of HPV infection that is considered as the main risk factor for carcinogenesis [30]. Moreover, T2DM is being redefined as an auto-immune disease [31–33], which may directly promote tumors [34–36]: B cell effects on glucose metabolism are linked to the production of pathogenic IgG antibodies [31]. Recently, an article has shown that long-term exposure to hyperinsulinemia in T2DM can lead immune T-cell to develop reduced sensitivity to its insulin receptors and result in impaired insulin signaling; therefore, these functional defect of insulin receptors on T cell may impose certain restrictions on antigen-specific T-cell immunity in T2DM [37]. There is another report that the presence of glutamic acid decarboxylase antibodies in latent autoimmune diabetes sub-groups in adults [33], has been associated with the development of multiple cancers [38]. In this study, we also found that after adjusting for the age, gender, alcohol use, and ASA co-morbidity score, T2DM had lower survival rate as compared to non-T2DM within the HPV-16 positive HNSCC groups. A previous preclinical study [39] revealed that prolonged glucose incubation or hyperglycemia could enhance HNSCC malignancy in time-course manner and be the reason behind increased resistance of DM to cisplatin chemotherapy agent. The authors emphasized on the requirement of tight glycemic control for clinical practice to achieve better prognostic outcomes for DM patients with HNSCC.

In our patients, HPV-16–associated HNSCC predominantly affected the tonsillar region, compared to the small percentage of tumors at the oral cavity. Our results are consistent with the previous findings that showed a higher prevalence of oncogenic HPV-16-DNA in palatine tonsils (50%) [40], compared to other HNSCC-tumor subtypes, such as oral cavity (12–18%), hypopharynx (13–25%), and larynx (3–7%) [41]. The reasons behind tonsils being more susceptible than other head and neck sites to HPV-16–DNA is unclear. It is known that tonsillar crypt epithelium capture and process antigens, which in turn facilitate viral access to the basal cells. It is possible that HPV-16–virus persists in the crypt epithelium and even in tonsillar lymphoid tissue, which makes the tonsillar tissue to be as HPV-16–reservoir in the head and neck regions [40]. It is also postulated that persistence of HPV-16–DNA in tonsillar lymphatic tissue could be of importance in the immune response to HPV-16 infection [40]; this cellular immune response is a key factor in the fight against HPV infections and related cancer. It can be the reason behind a better prognosis that we had seen in patients with HPV-positive tonsillar carcinoma as compared to non-tonsillar carcinoma.

Similar to any observational study, this investigation has a number of limitations. Although, through development of a series multivariate logistic regression models, the effect of observed confounders were adjusted, there might be a number of unobservable factors that could only be controlled with a randomized controlled trial. In addition, the limited sample size of this study could have resulted in some bias in the results produced. We also cannot exclude the possibility of associations between patient sexual behavior and HPV-16–positive HNSCC since we did not have access to this factor. Finally, this study was performed in one hospital, which may also limit the generalization of the results. Nonetheless, the data presented in this study are the first, to our knowledge, to suggest that T2DM is a prognostic factor for HPV-16 positive HNSCC, thus, these findings will need to be confirmed in a larger database, and the impact of preventive measures such as routine HPV-screening, testing for HPV, and even the prophylactic effects of HPV-vaccination on these high-risk groups be evaluated. Our findings could lead to further research about the impact of T2DM for promoting viral carcinogenesis and in deciphering the molecular mechanisms behind T2DM lower responsiveness to chemotherapy agents.

## Conclusions

In conclusion, our data show that history of primary HNSCC and occurrence of HNSCC in the tonsillar regions are patient-specific factors for HPV-positive HNSCC. We also were able to identify T2DM as a prognostic factor for HPV-positive HNSCC suggesting potential preventive strategy through HPV-16 screening of T2DM patients.

## Supporting information

S1 File Study raw data. (SAV)
